# Genome-Wide Identification of the PME Gene Family in Plum and Its Potential Roles in Fruit Texture Formation

**DOI:** 10.3390/genes17040469

**Published:** 2026-04-16

**Authors:** Longji Li, Yu Wang, Siyu Li, Yuan Wang, Menghan Wu, Yanke Geng, Gaopu Zhu, Danfeng Bai, Shaobin Yang, Fangdong Li, Taishan Li, Gaigai Du

**Affiliations:** 1 College of Horticulture and Plant Protection, Henan University of Science and Technology, Luoyang 471023, China; 12456agdf@gmail.com (L.L.);; 2 Research Institute of Non-Timber Forestry, Chinese Academy of Forestry, Zhengzhou 450003, China; 3 College of Food and Bioengineering, Henan University of Science and Technology, Luoyang 471023, China

**Keywords:** pectin methylesterase, plum, gene family, phylogeny, fruit texture, expression analysis

## Abstract

Background: Fruit texture is a major component of plum quality, affecting both consumer acceptance and postharvest behavior. Pectin methylesterases (PMEs) play important roles in cell-wall pectin modification and are therefore likely to contribute to plum fruit texture development and ripening-associated softening. However, the PME gene family has not yet been comprehensively investigated in plum (*Prunus salicina* L.). Methods: In the present study, a chromosome-level plum genome was used to survey this gene family at the whole-genome scale. Phylogenetic relationships, chromosomal positions, exon–intron organization, conserved motifs, domain architectures, gene duplication, and cis-elements were analyzed. Four flesh texture traits were measured in 55 plum accessions to characterize texture variation and select two representative cultivars with contrasting flesh textures for further molecular analysis. Based on the clustering results, ‘WSCL’ and ‘FR’ were selected for expression profiling during fruit development and subsequent correlation analysis with texture traits. Results: A total of 46 *PsPME* genes were identified. Phylogenetic analysis classified them into four major subgroups. Structural analyses indicated an overall conserved family framework, although noticeable variation was retained among individual members. Dispersed duplication made the largest contribution to family expansion, and most duplicated pairs appeared to have evolved under purifying selection. Correlation analysis showed that *PsPME20*, *PsPME22*, and *PsPME25* were significantly negatively correlated with flesh firmness, while *PsPME20* was additionally linked to flesh compactness and flesh fragility. Conclusions: Overall, this study clarifies the structural and evolutionary characteristics of the *PsPME* family and identifies candidate genes that may contribute to texture differences in plum, offering a basis for future functional studies and breeding programs.

## 1. Introduction

Plum (*P. salicina* L.) is a commercially important fruit crop cultivated worldwide, valued for its distinctive flavor and nutritional properties [1]. Fruit quality is largely determined by flesh texture, a key attribute that drives consumer preference and influences postharvest life, including storage potential and susceptibility to mechanical damage [2,3]. A major challenge for many commercial cultivars is the textural changes that occur during ripening and storage. This process reduces shelf life and increases the incidence of physical injuries and physiological disorders, resulting in significant economic losses [4,5]. Therefore, understanding the molecular basis of texture development and softening is essential for plum breeding.

Changes in fruit texture are closely associated with cell wall modification and alterations in intercellular adhesion [6]. Homogalacturonan (HG) is a major pectic polysaccharide in fleshy fruit cell walls, and its degree and pattern of methylesterification contribute to tissue mechanical properties [7,8]. Pectin methylesterases (PMEs) catalyze HG demethylesterification and generate negatively charged carboxyl groups that can modify the accessibility of HG to polygalacturonases and pectate lyases [9,10]. Thus, PME-mediated remodeling can both drive fruit softening and maintain local cell wall stability.

Evidence from stone fruits and other fleshy fruits indicates that PME contributes to texture regulation through multiple mechanisms. One major role of PME is to act early in pectin remodeling by modifying homogalacturonan properties and thereby affecting its accessibility to downstream polygalacturonases and pectate lyases. In apricot, PME activity increases at the color change stage, and PME-mediated modification has been proposed to prepare pectic substrates for polygalacturonase (PG), supporting an early role in fruit softening [11]. PME is also involved in the integration of postharvest signals, particularly under different temperature regimes. In peach, low-temperature storage suppresses PG, pectate lyase (PL), and PME activities and is accompanied by increased expression of C-repeat binding factor (CBF) genes, suggesting that cold-responsive signaling restrains pectin degradation and helps maintain firmness during storage [12]. By contrast, temperature fluctuations during cold-chain transport induce *PaPME3*, *PaPME4*, and *PaPG1–PaPG3*, enhance PME and PG activities, and accelerate the breakdown of sodium carbonate-soluble pectin, thereby promoting softening [13]. In addition, different PME isoforms may influence specific texture attributes rather than firmness alone. In apple, *MdMYB44* increases fruit fragility through direct activation of MdPME3. Likewise, in tomato, strong reduction in PME activity causes a marked loss of tissue integrity during senescence but has only a limited effect on firmness during ripening [14,15]. In *Prunus*, chilling-induced mealiness is closely associated with abnormal pectin solubilization and depolymerization, as well as increased pectin gel formation, suggesting that the balance between pectin demethylesterification and subsequent hydrolysis is disrupted under chilling conditions [16,17].

Recent studies have provided new insights into the molecular basis of fruit development and ripening in plums. In Japanese plum, ethylene-related treatments have been associated with differential DNA methylation during ripening, suggesting that DNA methylation contributes to the regulation of this process [18]. More broadly, fruit development and ripening are governed by coordinated phytohormonal, metabolic, transcriptomic, and epigenetic regulation, together with extensive cell wall remodeling [19]. The use of combined high-coverage and low-coverage whole-genome sequencing has enabled the identification of phenological QTLs and candidate genes in Japanese plum, providing valuable genomic resources for further trait dissection and breeding [20].

Although PME gene families have been characterized in related *Prunus* species, their specific roles in plum texture variation remain poorly understood [21]. A key gap is the lack of a genome-wide PME dataset for *P. salicina* that integrates family classification, duplication patterns, and structural characteristics. At the phenotypic level, plum texture evaluation has largely focused on a limited set of traits, while attributes such as flesh compactness and fragility have received considerably less attention [22,23]. Furthermore, studies that combine population-scale texture phenotyping with association analysis based on gene expression across fruit developmental stages remain scarce. This limits the efficient identification of candidate genes for plum breeding.

We combined genome-wide analysis of the *PsPME* gene family with multi-trait phenotyping to investigate its role in plum texture variation. A complete *PsPME* dataset was assembled, including information on chromosomal locations, phylogeny, duplication events, gene structures, and regulatory features. Natural variation in four flesh texture traits was evaluated in 55 plum accessions. Contrasting cultivars were then selected for expression analysis during fruit development, and correlation analysis identified candidate genes associated with texture. These findings provide a foundation for functional studies and offer practical resources for texture improvement and marker-assisted breeding in plum.

## 2. Materials and Methods

### 2.1. Genome-Wide Identification of the PsPME Gene Family in Plum

Genome-wide identification of the pectin methylesterase (PME) gene family was performed using the genome of *P. salicina* L. The genome assembly (FASTA) and genome annotation (GFF3) files were downloaded from the Genome Database for Rosaceae (https://www.rosaceae.org/Analysis/9450778, accessed on 5 July 2025) [24]. Protein sequences of *Arabidopsis thaliana* PMEs were retrieved from TAIR (release TAIR10) and used as queries for homology-based searches.

A combination of HMMER and BLASTp searches was used to identify putative *PsPME* proteins [25,26]. The hidden Markov model (HMM) profile of the Pectinesterase catalytic domain (Pfam: PF01095; release 37.1) was used to search the predicted plum proteome with HMMER (v3.4) using an E-value cutoff of $1\times 10^{-5}$. In parallel, PME protein sequences from *A. thaliana* were used as queries for BLASTp searches against the same dataset using BLAST+ (v2.17.0), with thresholds of E-value $\leq 1\times 10^{-5}$, sequence identity ≥30%, and query coverage ≥70%. Candidate proteins identified by both approaches were merged, and redundant sequences were removed.

Candidate proteins were further validated using the NCBI Conserved Domain Database (CDD, v3.21) and SMART (v10) [27,28]. Only proteins containing a complete Pectinesterase catalytic domain (PF01095) were retained as *PsPME* family members, whereas sequences with truncated catalytic domains or lacking conserved catalytic residues were excluded. Both proteins containing only the PME catalytic domain and those harboring an N-terminal PME inhibitor-like domain together with a complete PME catalytic domain were retained.

### 2.2. Physicochemical Characterization and Subcellular Localization Prediction of PsPME Proteins

Physicochemical properties of *PsPME* proteins, including amino acid length, molecular weight, theoretical isoelectric point, instability index, and grand average of hydropathicity, were calculated using the ProtParam module in Biopython (v1.86) [29]. Analyses were conducted using the final non-redundant protein set, in which only the longest isoform per gene locus was retained for downstream analyses to avoid redundancy. Subcellular localization was predicted from full-length protein sequences using DeepLoc (v2.0) [30].

### 2.3. Phylogenetic Analysis of PME Proteins in Representative Prunus Species

To investigate the evolutionary relationships of the PME family, protein sequences were collected from five *Prunus* species, including plum (*P. salicina*), sweet cherry (*P. avium*, https://www.rosaceae.org/Analysis/9262820, accessed on 5 July 2025), peach (*P. persica*, https://www.rosaceae.org/Analysis/24764266, accessed on 5 July 2025), Siberian apricot (*P. sibirica*, https://www.rosaceae.org/Analysis/9955981, accessed on 5 July 2025), and almond (*P. dulcis*, https://www.rosaceae.org/Analysis/20220996, accessed 5 on July 2025). For these species, PME family members were identified following the same pipeline as for *P. salicina*, in which candidate proteins were first retrieved by sequence similarity searches and subsequently retained only if they contained the conserved PME catalytic domain. The validated PME protein sequences were then aligned using MUSCLE (v5.1.0) [31], and poorly aligned regions were removed with trimAl (v1.5.1) using the “-automated1” option [32]. An approximate maximum-likelihood phylogenetic tree was constructed from the refined alignment using IQ-TREE (v2.3.6) [33], with branch support assessed by 1000 ultrafast bootstrap replicates. The final tree was visualized in R (v4.5.2) using the treeio (v1.34) and ggtree (v4.0.4) packages.

### 2.4. Gene Structure, Domain Architecture, and Conserved Motif Analysis of PsPME Genes

Gene structure features, including exon–intron organization, exon number, and arrangement patterns, were extracted from the plum genome annotation file (GFF3 format). For comparative analysis among subgroups, exon–intron structures were visualized together with the phylogenetic tree. Protein domain architectures were identified using the NCBI Conserved Domain Database (CDD, v3.21) and SMART (v10) and were examined across phylogenetic clades. Conserved motifs in full-length *PsPME* protein sequences were identified using MEME (v5.5.9), with the maximum number of motifs set to 10 and other parameters kept at their default settings [34]. An integrated visualization of the phylogenetic tree, gene structures, domain compositions, and motif distributions was generated in R (v4.5.2) using the ggtree (v4.0.4), ggplot2 (v4.0.1), and aplot (v0.2.9) packages.

### 2.5. Genes Duplication Pattern, Ka/Ks Estimation, and Synteny Analysis of PME Genes

To investigate the expansion pattern of the *PsPME* gene family in plum, duplication analysis was conducted using DupGen_finder (v1.0.0), which incorporates the MCScanX algorithm for duplication classification [35]. Duplicated genes were classified into five types: whole-genome duplication (WGD), tandem duplication (TD), proximal duplication (PD), dispersed duplication (DSD), and transposed duplication (TRD). For the identified duplicated *PsPME* gene pairs, nonsynonymous substitution rates (Ka), synonymous substitution rates (Ks), and Ka/Ks ratios were estimated using ParaAT (v2.0) [36], with protein sequences first aligned by ClustalW2 (v2.1) [37] and then converted into codon alignments.

To evaluate the evolutionary conservation and divergence of PME genes across *Prunus* species, comparative synteny analysis was conducted using plum (*P. salicina*, Psa) as the reference genome. Pairwise collinearity analyses were performed between Psa and four representative *Prunus* species, including sweet cherry (*P. avium*, Pav), peach (*P. persica*, Ppe), Siberian apricot (*P. sibirica*, Psi), and almond (*P. dulcis*, Pdu). PME genes in the other four *Prunus* species were identified using the same pipeline as that applied to *PsPME*. Based on these datasets, homologous gene pairs between *P. salicina* and each of the other four *Prunus* species were inferred through sequence similarity searches. Syntenic blocks were then identified using MCScanX based on sequence homology and genomic positional information. PME genes located within these syntenic blocks were subsequently extracted to infer putative orthologous relationships and to evaluate the conservation of corresponding loci across the compared genomes. The results were visualized in R (v4.5.2), with chromosomal distributions and Ka/Ks statistics plotted using ggplot2 (v4.0.1), syntenic relationships and duplication events were displayed as Circos plots using circlize (v0.4.17), and multi-panel figures were assembled using aplot (v0.2.9).

### 2.6. Cis-Element and Transcription Factor Binding Site Analysis of PsPME Gene Promoters

To analyze putative promoter regulatory features of *PsPME* genes in plum, the annotated 5′ end of the representative transcript retained for each locus (the longest isoform) was used as an annotation-based proxy for the transcription start site (TSS). Promoter regions were defined as the 2 kb upstream sequences relative to the proxy TSS and were extracted with BEDTools (v2.30.0) in a strand-aware manner according to transcript orientation. When the upstream interval was shorter than 2 kb because of scaffold or chromosome boundaries, the longest available upstream sequence was retained [38]. Cis-acting elements in *PsPME* promoters were predicted using PlantCARE [39], and the predicted elements were summarized by element type across promoters. Transcription factor binding sites were scanned with FIMO (MEME Suite v5.5.3) [40] using the CIS-BP Plants v1.0 motif library with a threshold of *p* ≤ 1 × 10^−5^, and the predicted sites were summarized by transcription factor family and site number across *PsPME* promoters for comparative analysis and visualization. All cis-element and transcription factor binding site (TFBS) results were integrated and visualized in R (v4.5.2). Phylogenetic trees were rendered with ggtree (v4.0.4), cis-element and TFBS annotation tracks were generated with ggplot2 (v4.0.1), and multi-panel figures were assembled using aplot (v0.2.9).

### 2.7. Fruit Texture Phenotyping of 55 Plum Germplasm Accessions

A total of 55 plum germplasm accessions were included for fruit texture phenotyping. All plant materials were cultivated under a uniform field management regime at the Yuanyang Experimental Station, Chinese Academy of Forestry (Yuanyang County, Henan, China; 35°04′ N, 113°58′ E). Fruits were collected at commercial maturity, and detailed information on the 55 plum accessions is provided in Supplementary Table S3. To minimize variation associated with differences in fruit maturity and sample heterogeneity, only healthy fruits with uniform external appearance, comparable size and color, and no visible symptoms of pests, diseases, or mechanical injury were selected for analysis.

For each accession, three healthy and representative trees grown under the same field conditions were selected as biological replicates, and six fruits were randomly harvested from each tree for texture evaluation. To reduce spatial variation within individual fruits, six evenly distributed and undamaged sites in the equatorial region of each fruit were subjected to puncture testing. The values obtained from the six puncture sites were averaged to generate a single texture value for each fruit. After harvest, the samples were transported to the laboratory and allowed to equilibrate at room temperature for 2 h. Surface moisture was removed immediately before measurement, and all texture measurements were completed within 6 h after harvest to minimize potential bias associated with short-term postharvest storage.

Fruit texture was assessed by a peeled puncture assay using a TA.XT Plus texture analyzer (Stable Micro Systems, Godalming, UK) equipped with a 2 mm cylindrical flat-ended probe and a 30 kg load cell. The test speed was set to 10 mm/s, the trigger force to 25 g, and the penetration depth to 4 mm. Force–displacement curves were recorded using Texture Exponent 7.0 software. Based on the instrument output, four texture-related parameters describing flesh mechanical properties were obtained for comparative analysis, including flesh firmness, flesh fragility, flesh compactness, and flesh fiber index.

These data were analyzed and visualized in R (v4.5.2). To classify the 55 plum accessions according to flesh mechanical properties, accession-level trait values were standardized by Z-score transformation and subjected to hierarchical clustering using Euclidean distance and Ward’s minimum variance method [41]. The standardized trait matrix together with the corresponding clustering dendrogram was visualized using ComplexHeatmap to illustrate similarities and differences in fruit texture among accessions. In addition, distribution plots of individual texture traits were generated using ggplot2 (v4.0.2).

### 2.8. RNA Extraction and qRT-PCR Analysis

To investigate the expression patterns of *PsPME* genes during plum fruit development, qRT-PCR analysis was performed using two representative cultivars with clearly contrasting flesh textures, selected based on the fruit texture phenotyping results obtained from the 55 plum germplasm accessions described above. These two cultivars were the firm-fleshed cultivar ‘Wushancuili’ (WSCL) and the soft-fleshed cultivar ‘Fengweimeigui’ (FR). Fruit samples were collected at three developmental stages, namely S1 (cell division stage), S2 (fruit expansion stage), and S3 (ripening stage). Stage assignment was based on published phenological information and the actual fruit developmental characteristics of the two cultivars under field conditions. Accordingly, fruit samples were collected at specific representative time points defined by days after full bloom (DAFB). In the firm-fleshed cultivar ‘Wushancuili’ (WSCL), samples were collected at 15 DAFB for S1 (cell division stage), 55 DAFB for S2 (fruit expansion stage), and 90 DAFB for S3 (ripening stage). In the soft-fleshed cultivar ‘Fengweimeigui’ (FR), the corresponding sampling points were 12 DAFB for S1, 45 DAFB for S2, and 78 DAFB for S3, respectively. For each cultivar at each developmental stage, three biological replicates were collected. Flesh tissues were immediately frozen in liquid nitrogen after sampling and stored at −80 °C until RNA extraction.

Total RNA was extracted using TRIzol reagent (TransGen Biotech, Beijing, China) according to the manufacturer’s instructions. RNA concentration and purity were assessed using a NanoDrop 2000 spectrophotometer (Thermo Fisher Scientific, Waltham, MA, USA), and RNA integrity was checked by agarose gel electrophoresis. Genomic DNA contamination was removed by DNase I treatment (Solarbio, Beijing, China). No-template controls and no-reverse-transcription controls were included to monitor reagent contamination and potential genomic DNA carryover. First-strand cDNA was synthesized from 1 μg of total RNA using the PrimeScript RT reagent Kit (TaKaRa, Dalian, China) at 37 °C for 15 min, followed by 85 °C for 5 s. Quantitative PCR was performed using 2× SYBR Green qPCR Premix (Universal) (Beijing Koton Biotechnology Co., Ltd., Beijing, China) on a LightCycler 480 II Real-Time PCR System (Roche Diagnostics, Basel, Switzerland). The amplification program consisted of an initial denaturation at 95 °C for 30 s, followed by 35 cycles of 95 °C for 10 s, primer-specific annealing for 10 s, and 72 °C for 30 s. Melting-curve analysis was performed after amplification to confirm product specificity.

qRT-PCR assays were performed for all candidate *PsPME* genes included in the expression analysis. Genes were then screened according to amplification quality, Ct values, and expression divergence between the two cultivars across developmental stages. Genes showing weak or unstable amplification signals (Ct > 35), limited expression divergence, particularly at S2 and S3 (fold change < 2.0), were not retained for detailed presentation in the main text. Based on these criteria, 15 genes were finally selected for further analysis and discussion, whereas the quantitative results for the remaining genes are shown in Figure S1.

Gene-specific primers were designed using Primer-BLAST and are listed in Table S1. *ACT2* was used as the internal reference gene [42]. Each biological replicate was analyzed with three technical replicates, and the mean Ct value was used for subsequent calculations. When technical replicates did not meet the preset consistency criteria, outlying values were excluded or the assay was repeated. Relative expression levels were first calculated using the 2^−ΔΔ*Ct*^ method. To facilitate comparison of expression patterns across samples and developmental stages, the mean expression value for each gene was calculated based on three biological replicates and three technical replicates for all samples, and the highest mean value of that gene was set to 1, with the expression levels of the remaining samples expressed relative to this maximum value. For statistical analysis, expression values derived from the 2^−ΔΔ*Ct*^ calculation were log2-transformed before hypothesis testing. At each developmental stage, expression differences between ‘WSCL’ and ‘FR’ were evaluated for each gene using a two-sided Welch’s *t*-test based on three biological replicates. The resulting *p*-values were adjusted across all gene-by-stage comparisons using the Benjamini–Hochberg method to control the false discovery rate, and adjusted *p* < 0.05 was considered statistically significant.

### 2.9. Correlation Analysis Between PsPME Expression and Fruit Texture Traits

Pearson correlation analysis was performed to evaluate the relationships between *PsPME* expression and fruit texture traits across three developmental stages (S1–S3) using R (v4.5.2). The analysis included two plum cultivars with contrasting flesh textures, ‘WSCL’ and ‘FR’, with three biological replicates collected for each cultivar at each developmental stage, resulting in a total of 18 samples (2 cultivars × 3 stages × 3 biological replicates; *n* = 18). For each gene–trait pair, the Pearson correlation coefficient (r) and corresponding two-sided *p*-value were calculated using the stats::cor.test() function. To account for multiple testing across all gene–trait combinations, *p*-values were adjusted using the Benjamini–Hochberg method, and the adjusted *p*-values were used to control the false discovery rate. Correlations with adjusted *p*-values < 0.05 were considered statistically significant. The correlation matrix was visualized using the corrplot package (v0.95).

## 3. Results

### 3.1. Identification of PsPME Family Members and Physicochemical Characterization of PsPME Proteins

To systematically identify members of the pectin methylesterase (PME) gene family in the *P. salicina* genome, a genome-wide analysis was performed using two complementary approaches. First, PME protein sequences from *A. thaliana* were used as queries for BLASTP searches. The candidate sequences were then subjected to hidden Markov model (HMM) screening using the Pfam pectinesterase catalytic domain profile (PF01095). After domain confirmation and removal of redundant transcripts, 46 high-confidence PME genes were identified and designated *PsPME1* to *PsPME46* according to their physical positions on the chromosomes (Table S2).

The basic physicochemical properties of the predicted *PsPME* proteins are summarized in Table S2. Protein length ranged from 94 to 595 aa, molecular weight (MW) ranged from 10.35 to 66.05 kDa, theoretical isoelectric point (pI) ranged from 4.63 to 10.48, grand average of hydropathicity (GRAVY) ranged from −0.66 to 0.14, and instability index (II) ranged from 5.70 to 51.61. Most *PsPME* proteins were predicted to be hydrophilic (44/46, GRAVY < 0) and stable (42/46, II < 40). The wide range of predicted pI values suggests substantial biochemical diversity within this gene family. In addition, subcellular localization prediction indicated that most *PsPME* proteins (34/46) were targeted at the cell wall.

### 3.2. Phylogenetic Classification of PME Proteins in Prunus

**Figure 1:**
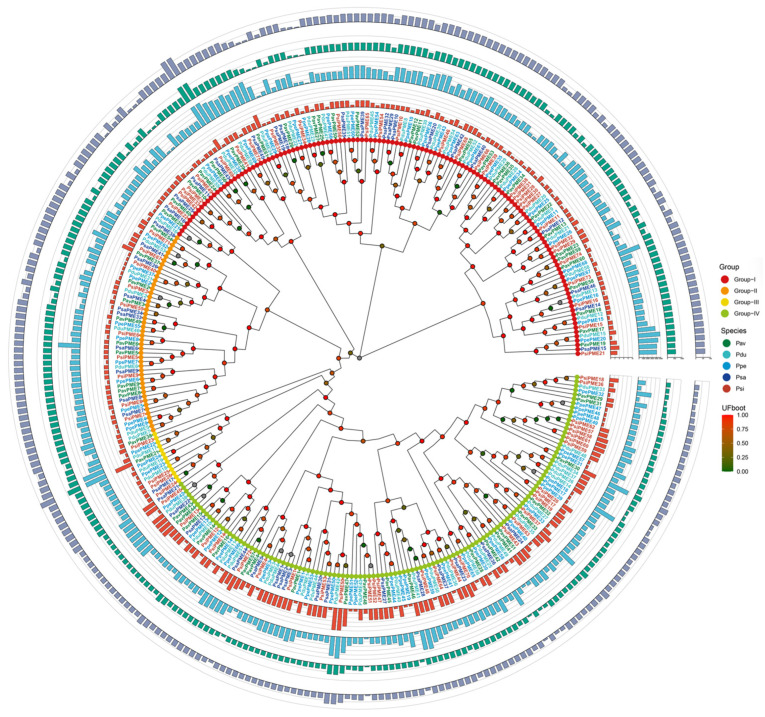
Circular maximum-likelihood phylogenetic tree of PME proteins from five *Prunus* species with integrated structural annotations. The phylogenetic tree was constructed based on the multiple sequence alignment of PME protein sequences from *P. sibirica* (Psi), *P. salicina* (Psa), *P. persica* (Ppe), *P. dulcis* (Pdu), and *P. avium* (Pav). PME proteins were classified into four major groups (Groups I–IV) according to tree topology and branch support values. Colored gene labels indicate species identity, and colored dots at the nodes represent ultrafast bootstrap support values. The four concentric bar tracks from inner to outer indicate exon number, gene length, coding sequence length, and predicted protein molecular weight, respectively.

To investigate the phylogenetic relationships of PME proteins in representative *Prunus* species, a circular maximum-likelihood tree was constructed based on the multiple sequence alignment of protein sequences (Figure 1). A total of 310 PME proteins from five *Prunus* species were included, namely *P. sibirica* (Psi, 75), *P. salicina* (Psa, 46), *P. persica* (Ppe, 69), *P. dulcis* (Pdu, 59), and *P. avium* (Pav, 61). According to the tree topology and branch support values, these proteins were classified into four major clades (Groups I–IV). Among them, Groups I and IV contained the largest numbers of PME proteins, with 128 and 123 members, respectively, followed by Group II with 47 members, whereas Group III was the smallest clade, containing only 12 members. Most internal branches showed moderate to strong support. In addition, cross-species comparison of the outer rings revealed clear differences among PME genes in gene length, exon number, CDS length, and predicted molecular weight.

### 3.3. Gene Structure, Domain Architecture, and Conserved Motif Features of the PsPME Family

**Figure 2:**
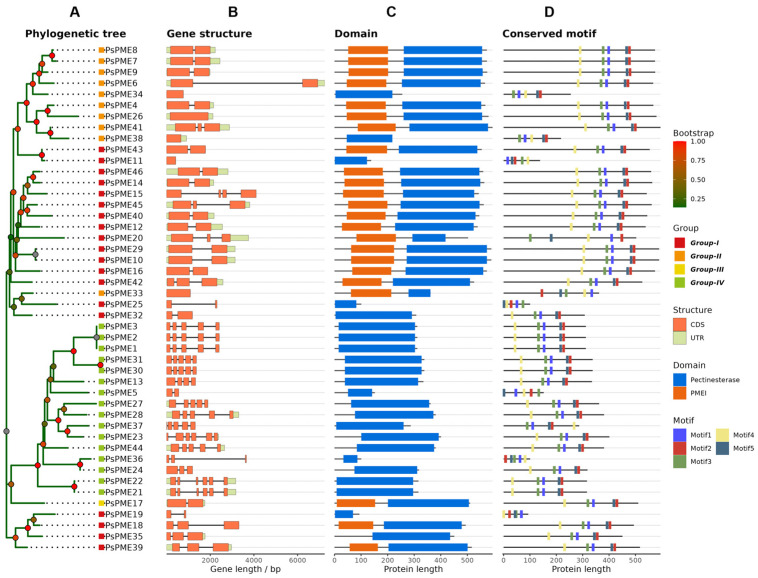
Phylogenetic relationships, gene structures, domain architectures, and conserved motifs of the *PsPME* gene. (**A**) Phylogenetic tree of 46 *PsPME* proteins. Colored labels indicate the four phylogenetic groups (Groups I–IV), and node colors represent bootstrap support values. (**B**) Exon–intron organization of *PsPME* genes. Orange boxes indicate coding sequences, light green boxes indicate untranslated regions, and black lines indicate introns. (**C**) Domain architecture of *PsPME* proteins. Blue boxes represent the Pectinesterase (PME) domain, and orange boxes represent the Pectin methylesterase inhibitor (PMEI) domain. (**D**) Distribution of conserved motifs identified in *PsPME* proteins. Different colored boxes represent different conserved motifs (Motifs 1–5).

To further explore the structural features of the *PsPME* gene family, we performed an integrated analysis of phylogenetic relationships, gene structures, protein domain architectures, and conserved motifs (Figure 2). The results showed that *PsPME* genes contained 1–6 exons, with genomic lengths ranging from 416 to 7241 bp. Among them, *PsPME6* was the longest gene, whereas *PsPME21*, *PsPME22*, and *PsPME23* had the highest exon numbers. Domain annotation indicated that *PsPME* proteins could be classified into two major types: proteins containing only the Pectinesterase (PME) domain (PF01095) and proteins containing both the Pectin methylesterase inhibitor (PMEI) domain (PF04043) and the PME domain (Figure 2C). The distribution of domain architectures varied among phylogenetic groups: Group IV members contained only the PME domain, whereas Groups I and II were enriched in dual-domain (PMEI + PME) proteins. Notably, the single Group III member also harbored both PMEI and PME domains (Figure 2C). Conserved motif analysis identified five major motifs (Motifs 1–5), and most members showed similar motif compositions and arrangement patterns (Figure 2D).

### 3.4. Chromosomal Distribution, Gene Duplication, and Selective Pressure Analysis of PsPME Genes

**Figure 3:**
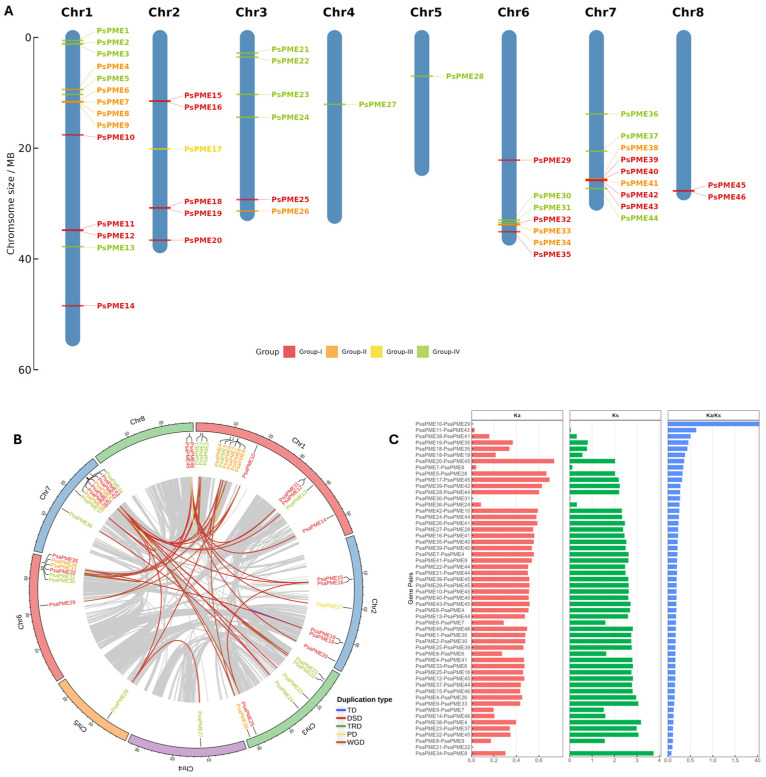
Chromosomal distribution, duplication patterns, and sequence divergence of *PsPME* gene family. (**A**) Chromosomal distribution of the 46 *PsPME* genes across the eight chromosomes of *P. salicina*. Gene names are colored according to phylogenetic group classification (Groups I–IV). (**B**) Circos plot shows the chromosomal locations and duplication relationships of *PsPME* genes. Colored links indicate different duplication types. Gray lines represent genome-wide collinear relationships in the background. (**C**) Ka, Ks, and Ka/Ks values of duplicated *PsPME* gene pairs. Each bar represents one duplicated gene pair identified in the *PsPME* family.

To characterize the chromosomal distribution patterns of the *PsPME* gene family, the 46 *PsPME* genes were mapped to the eight chromosomes of *P. salicina* (Figure 3A). The *PsPME* genes showed an uneven chromosomal distribution. Chromosome 1 contained the largest number of genes (14), followed by Chromosome 7 (9) and Chromosome 6 (7). Chromosomes 2 and 3 each harbored six genes, Chromosome 8 contained two genes, and only one gene was identified on Chromosomes 4 and 5. In addition, several clusters of adjacent genes were observed, including *PsPME6*–*PsPME9* on Chromosome 1, *PsPME32*–*PsPME34* on Chromosome 6, and *PsPME38*–*PsPME43* on Chromosome 7 (Figure 3A).

Duplication patterns of *PsPME* genes were further evaluated together with collinearity relationships (Figure 3B). A total of 57 duplicated gene pairs were detected, including 41 dispersed duplication (DSD) pairs (71.9%), 7 whole-genome duplication (WGD) pairs (12.3%), 5 tandem duplication (TD) pairs (8.8%), and 4 proximal duplication (PD) pairs (7.0%). No transposed duplication (TRD) events were detected. These results indicate that DSD was the predominant duplication mode contributing to the expansion of the *PsPME* gene family.

Furthermore, the nonsynonymous substitution rate (Ka), synonymous substitution rate (Ks), and Ka/Ks ratio were calculated for duplicated gene pairs (Figure 3C). Three gene pairs with identical coding sequences (Ka = 0, Ks = 0) yielded an undefined Ka/Ks ratio.

Among the remaining 54 duplicated gene pairs, 53 exhibited Ka/Ks values below 1 (0.081–0.632), indicating that most duplicated *PsPME* gene pairs evolved under purifying selection. One gene pair, *PsPME10*–*PsPME29*, showed an exceptionally high Ka/Ks value of 43.8. This estimate ratio resulted from an extremely low Ks value (0.0000869) and should not be interpreted as reliable evidence of positive selection.

### 3.5. Cross-Species Synteny Analysis of PME Gene Family Among Five Prunus Species

**Figure 4:**
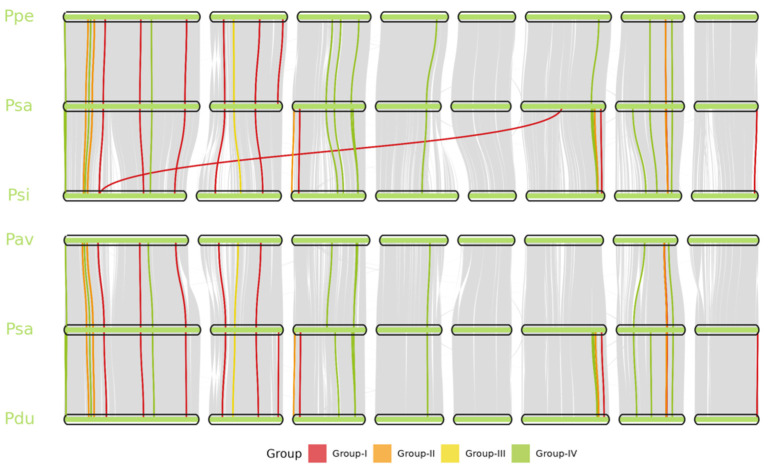
Cross-species synteny analysis of *PsPME* genes among five *Prunus* species. Horizontal segments represent chromosomes of *P. salicina* (Psa), *P. avium* (Pav), *P. persica* (Ppe), *P. sibirica* (Psi), and *P. dulcis* (Pdu). Gray ribbons show genome-wide synteny, and colored links mark syntenic *PsPME* gene pairs. Link colors indicate phylogenetic groups (Groups I–IV).

To assess the evolutionary conservation of *PME* genes among *Prunus* species, cross-species synteny analysis was performed using *P. salicina* (Psa) as the reference and compared with *P. persica* (Ppe), *P. sibirica* (Psi), *P. avium* (Pav), and *P. dulcis* (Pdu) (Figure 4). A total of 28, 26, 30, and 25 syntenic *PME* gene pairs were identified in the Psa–Ppe, Psa–Psi, Psa–Pav, and Psa–Pdu comparisons, respectively. In total, 29 unique Psa*PME* genes were involved in at least one cross-species collinear relationship. The syntenic gene pairs were mainly distributed in Groups I and IV, whereas Group III contained the fewest collinear pairs. Among the four pairwise comparisons, the Psa–Pav comparison showed the highest number of syntenic *PME* gene pairs.

### 3.6. Cis-Acting Regulatory Elements and Predicted Transcription Factor Binding Sites in PsPME Promoters

**Figure 5:**
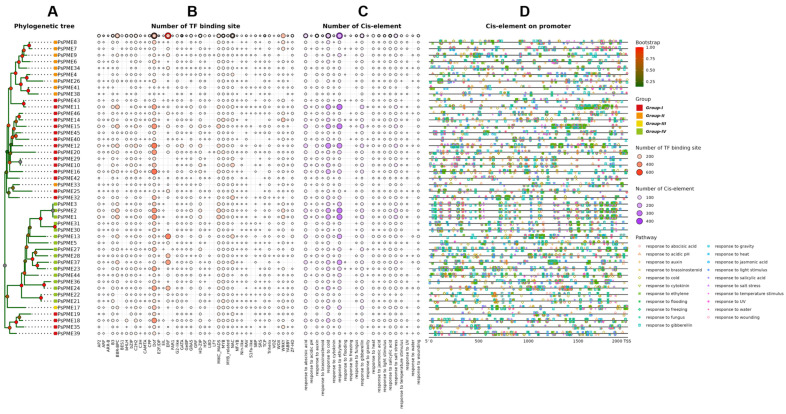
Cis-acting regulatory elements and predicted transcription factor binding sites in the promoters of *PsPME* genes. (**A**) Phylogenetic tree of 46 *PsPME* proteins. Colored labels represent the four phylogenetic groups (Groups I–IV). (**B**) Bubble plot showing the number of predicted transcription factor binding sites (TFBSs) from different transcription factor families in the 2 kb upstream regions of *PsPME* genes. Bubble size and color represent the number of predicted TFBSs. (**C**) Bubble plot showing the number of cis-acting elements belonging to different functional categories in the 2 kb upstream promoter regions of *PsPME* genes. Bubble size represents the number of cis-acting elements. (**D**) Distribution of cis-acting regulatory elements along the 2 kb upstream promoter regions of *PsPME* genes. Different symbols and colors represent different functional categories of cis-acting elements.

To characterize the regulatory features of the *PsPME* gene family, the 2 kb upstream promoter regions of all 46 *PsPME* genes were analyzed for cis-acting elements and transcription factor binding sites (TFBSs) (Figure 5). A total of 9513 predicted TFBSs were identified, with the number per promoter ranging from 3 to 462 (Figure 5B). Among the transcription factor families, Dof motifs were the most abundant, accounting for 47.62% of all predicted TFBSs, followed by BBR-BPC (16.15%), B3 (8.50%), AP2 (8.42%), MIKC_MADS (8.04%), and GRAS (6.75%). The abundance of predicted TFBSs varied among phylogenetic groups, with Group IV showing the highest mean number of sites per promoter (264.75), whereas Group II showed the lowest (124.60) (Figure 5B).

PlantCARE analysis further identified 6910 cis-acting elements across the 46 *PsPME* promoters, with 51 to 289 elements detected in individual promoters (Figure 5C,D). Among the major functional categories, low-temperature-responsive elements (20.07%) and ethylene-responsive elements (19.23%) were the most abundant, followed by ABA-responsive (11.95%), gibberellin-responsive (10.97%), salt stress-responsive (8.73%), and auxin-responsive elements (8.62%). All ten major regulatory categories were represented in the *PsPME* promoter set, although their abundance varied among phylogenetic groups (Figure 5C).

The distribution map showed that both cis-acting elements and predicted TFBSs were broadly distributed throughout the 2 kb upstream regions rather than clustered within a limited interval (Figure 5D). These results indicate that *PsPME* promoters contain diverse regulatory features associated with hormone signaling, stress responsiveness, and transcriptional regulation.

### 3.7. Comprehensive Fruit Texture Profiling of 55 Plum Cultivars

**Figure 6:**
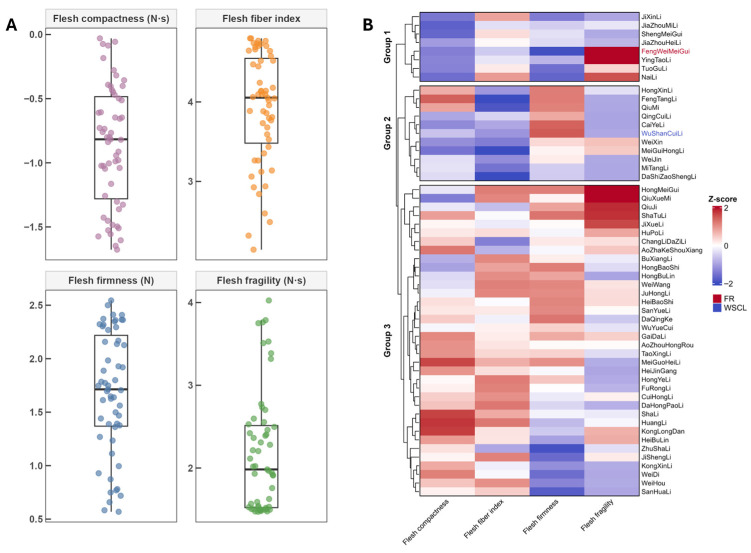
Comprehensive profiling of fruit texture traits in 55 plum cultivars. (**A**) Distribution of four texture-related parameters measured by puncture assay, including flesh compactness, flesh fiber index, flesh firmness, and flesh fragility. Each dot represents one cultivar, and boxplots summarize the overall distribution of each trait. (**B**) Hierarchical clustering heatmap of 55 plum cultivars based on Z-score-standardized fruit texture traits. Cultivars were grouped into three clusters according to Euclidean distance and the UPGMA method. Color intensity indicates the relative level of each trait after standardization. ‘Fengweimeigui’ (FR) and ‘Wushancuili’ (WSCL) are highlighted as representative cultivars with contrasting texture profiles.

Flesh texture variation was quantified in 55 plum cultivars using a puncture assay with a texture analyzer to support cultivar-wide phenotyping and the selection of representative materials for subsequent quantitative analyses. The phenotypic information for these accessions is summarized in Supplementary Table S3. An integrated assessment combined single-trait distributions and hierarchical clustering of standardized texture parameters (Figure 6).

Four texture-related parameters were extracted from the puncture assay, namely flesh firmness (N), flesh fragility (N·s), flesh fiber index (dimensionless), and flesh compactness (N·s) (Figure 6A). Flesh firmness ranged from 0.57 to 2.54 N, with a mean value of 1.69 N. Flesh fragility ranged from 1.47 to 4.03 N·s, with a mean value of 2.18 N·s. The flesh fiber index ranged from 2.14 to 4.80, with a mean value of 3.92. Flesh compactness exhibited a bimodal distribution across cultivars, ranging from −1.68 to −0.03 N·s, with a mean value of −0.86 N·s. Overall, these results indicate substantial difference in flesh mechanical properties among the 55 cultivars.

To compare overall texture patterns among cultivars, hierarchical clustering was performed using Z-score-standardized traits with Euclidean distance and the UPGMA method (Figure 6B). This analysis divided the 55 cultivars into three clusters with distinct texture characteristics. Cluster 1 contained 8 cultivars and generally showed low firmness, low fragility, and reduced compactness, whereas the fiber index varied within the group. Representative cultivars in this cluster included ‘Fengweimeigui’ (FR), ‘Yingtaoli’, and ‘Naili’. Cluster 2 included 11 cultivars and was characterized by intermediate overall trait levels, although clear variation was still present among accessions within the cluster. Representative cultivars in this group included ‘Wushancuili’ (WSCL), ‘Qingcuili’, and ‘Caili’. Cluster 3 contained 36 cultivars and generally exhibited higher firmness and fragility, with corresponding shifts in standardized trait patterns; representative cultivars included ‘Hongmeigui’, ‘Shatuli’, and ‘Hongbulin’. Clear contrasts in standardized texture patterns were observed between Clusters 1 and 2. On this basis, Fengweimeigui’ (FR; Cluster 1) and ‘Wushancuili’ (WSCL; Cluster 2) were selected as representative materials for subsequent analyses linking *PsPME* expression to divergent fruit texture types.

### 3.8. Developmental Expression Patterns of Candidate PsPME Genes in WSCL and FR Fruits

**Figure 7:**
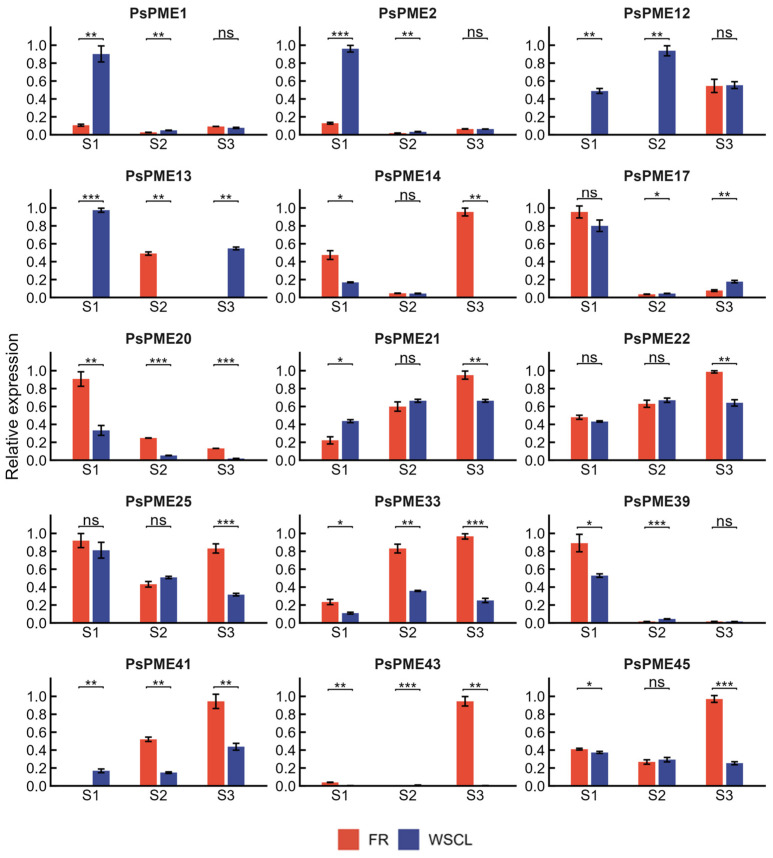
Expression profiles of 15 selected *PsPME* genes in two plum cultivars during fruit development. Relative expression levels of 15 selected *PsPME* genes were determined by qRT-PCR in the soft-fleshed cultivar ‘FR’ and the firm-fleshed cultivar ‘WSCL’ at three developmental stages, including S1 (cell division), S2 (fruit expansion), and S3 (ripening). Expression values for each gene were normalized to the maximum expression level across all samples and are presented as mean ± SD. Differences between ‘FR’ and ‘WSCL’ at each developmental stage were evaluated using a two-sided Welch’s *t*-test based on log2-transformed values, followed by Benjamini–Hochberg correction. ns, not significant; * FDR < 0.05; ** FDR < 0.01; *** FDR < 0.001.

The expression patterns of 15 selected *PsPME* genes were analyzed by qRT-PCR in two plum cultivars with contrasting flesh textures, ‘WSCL’ and ‘FR’, across three fruit developmental stages: S1 (cell division), S2 (fruit expansion), and S3 (ripening) (Figure 7). The expression profiles of these selected genes are shown in Figure 7, whereas the remaining PsPME family members are presented in Figure S1.

Transcript abundance varied considerably across developmental stages and between the two cultivars. *PsPME1*, *PsPME2*, and *PsPME12* showed higher expression levels in ‘WSCL’ at S1 and S2, whereas these differences largely disappeared at S3. *PsPME17* showed similar expression levels in the two cultivars at S1 but became more highly expressed in ‘WSCL’ at S2 and remained elevated through S3. In contrast, *PsPME20* and *PsPME33* maintained consistently higher expression levels in ‘FR’ across all three developmental stages. *PsPME39* also showed higher expression in ‘FR’ at S1 and S2, but its transcript level decreased to a low level in both cultivars at S3.

More obvious cultivar-specific differences were detected at the ripening stage. At S3, *PsPME14*, *PsPME22*, *PsPME25*, and *PsPME45* all showed significantly higher expression in ‘FR’, and *PsPME14* also showed higher expression in ‘FR’ at S1. Several genes displayed clear developmental shifts in expression patterns. *PsPME21* was more highly expressed in ‘WSCL’ at S1 but became predominantly expressed in ‘FR’ at S3. A similar pattern was observed for *PsPME41*, which showed higher expression in ‘WSCL’ at S1 and higher expression in ‘FR’ at S2 and S3. *PsPME43* showed a comparable trend, with preferential expression in ‘WSCL’ at S1 followed by marked up-regulation in ‘FR’ at later stages. *PsPME13* displayed a more complex pattern, with higher transcript abundance alternating between ‘WSCL’ (S1), ‘FR’ (S2), and ‘WSCL’ (S3).

Overall, these results indicate that the selected *PsPME* genes exhibit distinct stage-specific and cultivar-specific expression patterns during plum fruit development. Some genes showed more pronounced differences at early developmental stages, whereas others displayed stronger divergence during ripening. These findings suggest that different *PsPME* family members may contribute differently to texture formation in plum fruit and provide candidate genes for further functional analysis.

### 3.9. Correlation of Candidate PsPME Expression with Fruit Texture Traits During Plum Developmental Stage

**Figure 8:**
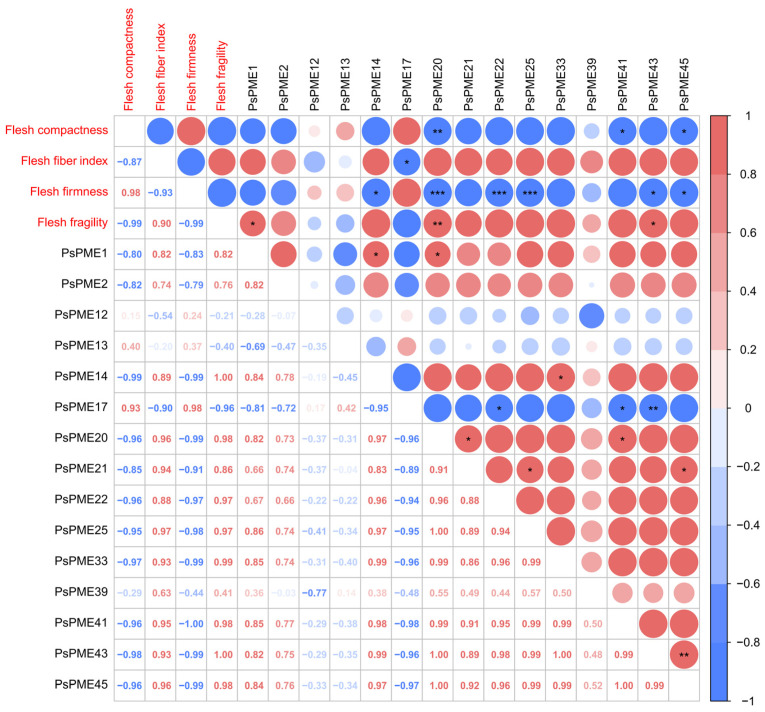
Pearson correlation analysis between *PsPME* expression and fruit texture traits at the developmental stage in two contrasting plum cultivars, ’WSCL’ and ‘FR’. The analysis was based on standardized values of four texture traits, including flesh compactness, flesh fiber index, flesh firmness, and flesh fragility, together with selected *PsPME* genes. Circle color indicates correlation direction (red, positive; blue, negative), and circle size reflects correlation strength. Correlation coefficients (r) are shown in the lower triangle, and circles are shown in the upper triangle. Asterisks indicate statistical significance (* FDR < 0.05, ** FDR < 0.01, *** FDR < 0.001).

To assess the relationship between *PsPME* expression and fruit texture during plum fruit development, Pearson correlation analysis was performed using samples from two plum cultivars with contrasting texture characteristics, ‘WSCL’ and ‘FR’, collected at three developmental stages (S1–S3), with three biological replicates per stage for each cultivar (total *n* = 18) (Figure 8). Four texture parameters were included in the analysis, namely flesh firmness, flesh fragility, flesh fiber index, and flesh compactness. Significant correlations were detected between several *PsPME* genes and fruit texture traits, with the strongest associations observed for flesh firmness. Notably, *PsPME20*, *PsPME22*, and *PsPME25* showed strong negative correlations with flesh firmness (*r* < 0, *p* < 0.001; Figure 8), indicating that higher expression of these genes was associated with lower flesh firmness. In addition, *PsPME20* showed significant correlations with multiple traits, including a negative correlation with flesh compactness and a positive correlation with flesh fragility (*p* < 0.05; Figure 8). These results suggest that *PsPME20* may play a broader role in the regulation of fruit texture in plum.

## 4. Discussion

PMEs have been widely involved in fruit texture regulation through their roles in pectin demethylesterification, which affects cell wall properties and contributes to softening during ripening [43]. In this study, genome-wide analysis identified 46 members of the *PsPME* family. For comparison, PME family members were also identified in four related *Prunus* species—*P. sibirica*, *P. persica*, *P. dulcis*, and *P. avium*—with 75, 69, 59, and 61 genes detected, respectively, indicating that plum possesses a relatively small PME family among the species examined. Phylogenetic analysis further showed that PME proteins from plum and the four related species clustered into four major conserved clades. Together, these findings indicate that the *PsPME* family is evolutionarily conserved but still exhibits structural and regulatory diversity that may underlie functional differentiation during plum fruit development.

Promoter analysis showed that *PsPME* genes contain abundant regulatory elements related to phytohormones, environmental signals, and abiotic stress responses, suggesting that these genes may respond to multiple cues associated with fruit ripening and texture formation. Gene duplication analysis further indicated that dispersed duplication represented the predominant mode of *PsPME* family expansion. Most duplicated gene pairs had Ka/Ks ratios below 1, consistent with purifying selection during evolution. One exception was the *PsPME10–PsPME29* pair, which showed an unusually high Ka/Ks value. This ratio was most likely inflated by an extremely low Ks value, pointing to a very recent duplication event [44]. Overall, these results suggest that although most *PsPME* genes have remained evolutionarily constrained, a few members may have undergone recent divergence that contributed to regulatory or functional specialization.

Fruit texture phenotyping across 55 plum cultivars revealed marked variation in four core mechanical traits, namely flesh firmness, flesh fragility, flesh fiber index, and flesh compactness. Hierarchical clustering of the standardized trait matrix separated the cultivars into three distinct texture types. A previous study on plum cultivars likewise classified the materials into distinct clusters on the basis of fruit texture data [45], supporting the use of mechanical phenotypes for texture classification. At the population level, cultivars with higher flesh firmness generally also showed coordinated changes in flesh fragility and other mechanical traits, indicating that these traits reflect linked variation in flesh mechanical properties. This phenotypic framework guided the selection of two representative cultivars with contrasting flesh textures, namely ‘WSCL’ (firm-flesh) and ‘FR’ (soft-flesh), for subsequent expression analysis.

qRT-PCR analysis of 15 selected *PsPME* genes revealed distinct stage- and cultivar-dependent expression profiles during fruit development in ‘WSCL’ and ‘FR’. *PsPME1*, *PsPME2*, and *PsPME12* showed relatively higher expression in ‘WSCL’ at early developmental stages, whereas these differences were less evident at the ripening stage. By contrast, *PsPME20* and *PsPME33* maintained consistently higher expression in ‘FR’ throughout development. Divergence between the two cultivars became more pronounced at S3, when *PsPME14*, *PsPME22*, *PsPME25*, and *PsPME45* all showed significantly higher expression in ‘FR’. In addition, *PsPME21*, *PsPME41*, and *PsPME43* exhibited marked shifts in cultivar-preferential expression across development. These patterns suggest that different *PsPME* family members may act at different stages of plum fruit development in a cultivar-dependent manner, with some contributing primarily at early stages and others playing a stronger role during ripening. This interpretation is consistent with the stage-dependent expression reported for PME genes during peach fruit ripening [46].

Correlation analysis further linked plum fruit texture variation to *PsPME* expression. Among the measured traits, flesh firmness showed the strongest associations with candidate gene expression, with *PsPME20*, *PsPME22*, and *PsPME25* being significantly negatively correlated with flesh firmness. In addition, *PsPME20* was positively correlated with flesh fragility, suggesting that this gene may be involved not only in firmness decline but also in fracture-related properties of the tissue. This interpretation is biologically plausible because pectin remodeling affects cell-to-cell adhesion, cell wall stiffness, and tissue integrity, all of which are central to the structural changes that accompany fruit softening. Comparable evidence has been reported in other fruit crops. In strawberry, genome-wide and functional analyses showed that PME-mediated cell wall remodeling is a key component of fruit softening, and *FvPME38* was identified as an important regulator of fruit firmness [47]. In European pear, *PcPME63* was identified as a key gene closely associated with post-cold-storage softening and firmness decline [48]. In peach, transcript profiling of PME and PME inhibitor (*PMEI*) family members also revealed clear ripening stage-dependent expression patterns, suggesting that different PME family members may contribute to texture formation at different stages of fruit development [49]. Collectively, these observations indicate that the correlations detected in plum are not isolated and support the view that PME-mediated pectin modification is broadly involved in softening and texture differentiation in fleshy fruits.

The present results therefore support the hypothesis that specific *PsPME* genes contribute to plum fruit softening and texture differentiation through pectin modification and cell wall remodeling during fruit development and ripening. Within this framework, flesh firmness appears to be the texture trait most strongly associated with *PsPME* activity, whereas *PsPME20* may also influence flesh fragility and fracture-related behavior. By integrating population-level phenotyping, cultivar-level expression profiling, and gene–trait association analysis, this study narrows the relatively large *PsPME* family to a smaller set of biologically meaningful candidate genes.

This study also has several limitations. The qRT-PCR analysis was conducted in only two contrasting cultivars, and the observed gene–trait correlations, although informative, do not by themselves establish causality. In addition, the effects of PME genes on fruit texture are likely influenced by multiple factors, including enzyme activity, pectin methylesterification patterns, calcium cross-linking, and interactions with other cell wall-modifying enzymes. Further work combining biochemical characterization with functional validation will be required to define the precise contributions of individual *PsPME* genes to plum fruit softening and texture differentiation.

From a breeding and production perspective, the candidate genes identified here, especially *PsPME20*, *PsPME22*, and *PsPME25*, represent promising targets for future functional validation, marker development, and germplasm evaluation. These genes may facilitate the selection of plum cultivars with improved flesh firmness, more desirable texture characteristics, and better postharvest performance. Thus, this study not only advances the biological interpretation of plum fruit texture formation but also provides a practical basis for texture-oriented plum breeding and postharvest quality improvement.

## 5. Conclusions

In this study, we identified 46 *PsPME* genes in plum (*P. salicina*) and characterized their phylogenetic relationships, structural features, duplication patterns, promoter cis-elements, and expression profiles. These analyses showed that the *PsPME* family is evolutionarily conserved in plum, while also retaining structural and regulatory diversity that may underlie functional differentiation among family members.

More importantly, our results support the biological hypothesis that specific *PsPME* genes contribute to plum fruit softening and texture differentiation by participating in pectin remodeling and cell wall modification during fruit development and ripening. Phenotypic evaluation of 55 plum accessions revealed substantial variation in texture-related traits and provided the basis for selecting two cultivars with contrasting flesh textures for expression analysis. By integrating developmental expression profiling with gene–trait correlation analysis, *PsPME20*, *PsPME22*, and *PsPME25* were prioritized as candidate genes associated with flesh firmness, while *PsPME20* also showed additional associations with flesh compactness and flesh fragility. These findings suggest that some *PsPME* members may influence not only firmness decline, but also other mechanical properties of plum flesh during ripening. Overall, this study provides a genome-wide resource for the *PsPME* gene family in plum and offers biologically meaningful candidate genes for future functional validation. From an applied perspective, these genes may serve as potential targets for molecular marker development, texture-oriented breeding, and postharvest quality improvement in plum.

## Data Availability

The original contributions presented in this study are included in the article or Supplementary Materials. Further inquiries can be directed at the corresponding author.

## Supplementary Materials

The following supporting information can be downloaded at: https://www.mdpi.com/article/10.3390/genes17040469/s1, Figure S1. qRT-PCR expression profiles of additional *PsPME* genes across three fruit developmental stages in ‘Wushancuili’ (WSCL) and ‘Fengweimeigui’ (FR); Table S1. Primer sequences used for qRT-PCR; Table S2. Physicochemical properties of *PsPME* proteins identified in plum; Table S3. The basic background and phenotypic information for the 55 plum accessions.
