# Supplementary figures and images for: Genome-Wide Identification of the PME Gene Family in Plum and Its Potential Roles in Fruit Texture Formation
Source: Genes (Basel). 2026 Apr 16;17(4):469. doi: 10.3390/genes17040469 (PMC13115721; doi:10.3390/genes17040469)

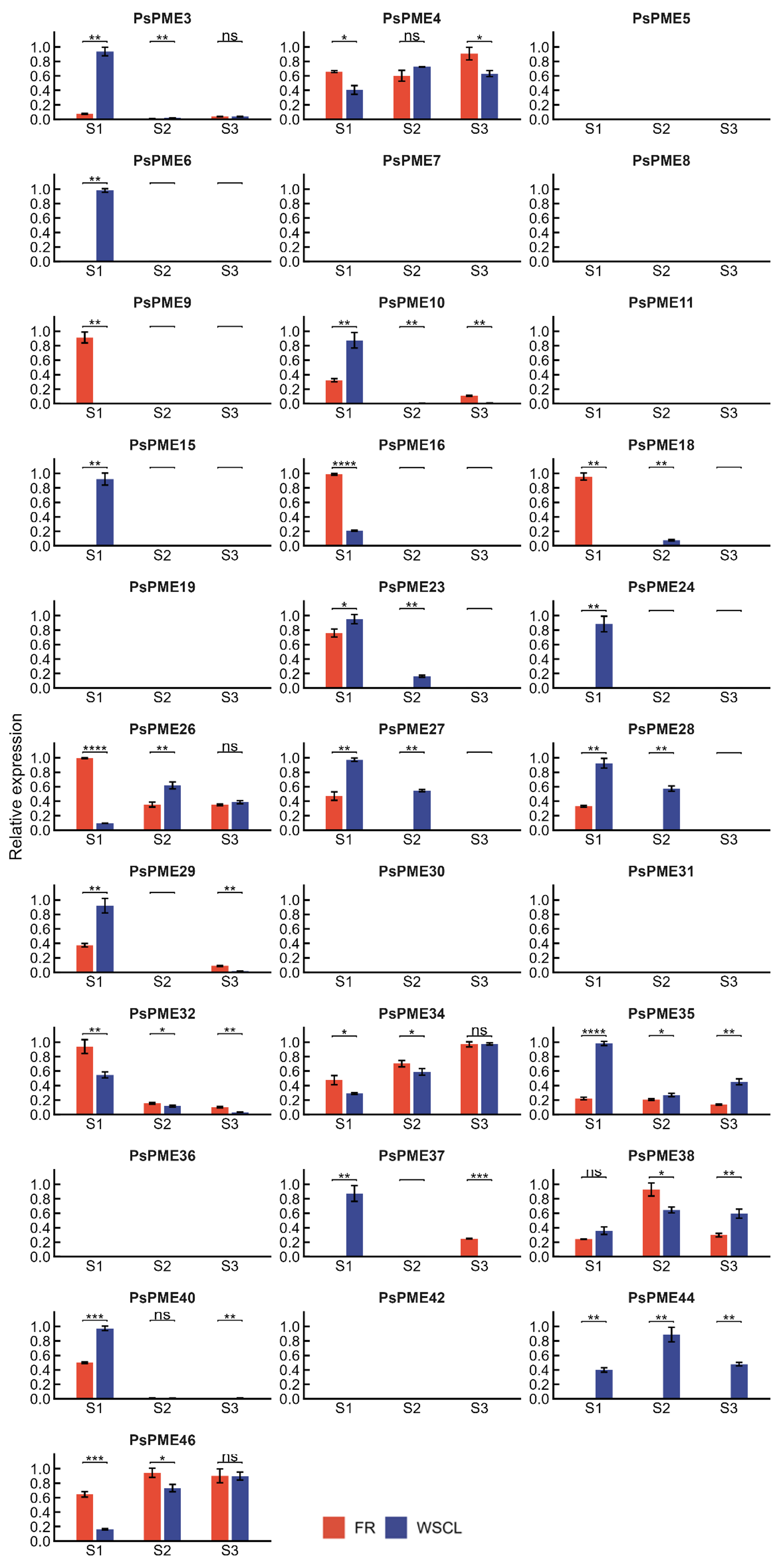

Supplement: Supplementary file 1 [file genes-17-00469-s001.zip › Supplementary Files/Figure S1.png]
